# Regulation of Adaptive Tumor Immunity by Non-Coding RNAs

**DOI:** 10.3390/cancers13225651

**Published:** 2021-11-12

**Authors:** Eleftheria Papaioannou, María del Pilar González-Molina, Ana M. Prieto-Muñoz, Laura Gámez-Reche, Alicia González-Martín

**Affiliations:** Department of Biochemistry, Universidad Autónoma de Madrid (UAM), Instituto de Investigaciones Biomédicas Alberto Sols (CSIC-UAM), 28029 Madrid, Spain; epapaioannou@iib.uam.es (E.P.); mdpgonzalez@iib.uam.es (M.d.P.G.-M.); amprieto@iib.uam.es (A.M.P.-M.); lgamez@iib.uam.es (L.G.-R.)

**Keywords:** tumor immunology, non-coding RNAs, microRNAs, long non-coding RNAs, cancer immunotherapy

## Abstract

**Simple Summary:**

Recent advances in basic tumor immunology have enabled the development of cancer immunotherapies. These therapies harness the natural capacity of our adaptive immune system to detect and eliminate tumor cells. While most research on the immune response to tumors has focused on protein-coding genes, the potential roles of non-coding RNAs (ncRNAs) in this process remain largely unexplored. In this review, we compile recent evidence on the ncRNA-mediated regulation of adaptive tumor immunity highlighting the need for further research in this field, and discuss their therapeutic potential to improve cancer immunotherapy.

**Abstract:**

Cancer immunology research has mainly focused on the role of protein-coding genes in regulating immune responses to tumors. However, despite more than 70% of the human genome is transcribed, less than 2% encodes proteins. Many non-coding RNAs (ncRNAs), including microRNAs (miRNAs) and long non-coding RNAs (lncRNAs), have been identified as critical regulators of immune cell development and function, suggesting that they might play important roles in orchestrating immune responses against tumors. In this review, we summarize the scientific advances on the role of ncRNAs in regulating adaptive tumor immunity, and discuss their potential therapeutic value in the context of cancer immunotherapy.

## 1. Introduction

Cancer is one of the main causes of mortality and morbidity worldwide. The Global Cancer Observatory (GCO) 2020 estimated 18.1 million new cases and 9.6 million cancer-related deaths, with an expected rise in incidence up to 27.5 million new cases worldwide by 2040 [1]. Cancer therapeutics have significantly progressed during the past few decades, but the current mortality rate remains high, which implies the need for developing new effective treatments for this disease.

Tumor development involves a series of sequential events starting with mutations in proto-oncogenes and tumor suppressor genes that lead to uncontrolled cell division and tumor generation. This is followed by angiogenesis and, in advanced stages, invasion and metastasis. The immune system plays a critical role during all phases of tumor development and, indeed, the cancer immunosurveillance concept states that the immune system recognizes and eliminates many arising tumors before they grow into detectable malignancies [2]. The initial mutations in tumor cells often lead to the expression of tumor neoantigens that, in many cases, are recognized by the adaptive immune system, comprised by T and B cells [3,4,5,6]. In addition, tumor growth and invasion produce tissue damage that activates the innate immune defenses which, in turn, recruit adaptive immunity that mounts antigen-specific responses against tumor cells. However, tumor cells with low-immunogenicity mutations are frequently ignored by the immune system and continue to proliferate [7,8,9]. Some tumors also produce immunosuppressive molecules such as transforming growth factor (TGF)-β or interleukin (IL)-10 [8,10,11], downregulate antigen presentation mechanisms, or activate negative regulatory checkpoints present in T lymphocytes, such as cytotoxic T lymphocyte antigen 4 (CTLA-4) or programmed cell death protein 1 (PD-1) to impair the antitumoral T cell response [11,12,13].

Our current understanding of the basic immunobiology of cancer has enabled the development of a series of immunotherapy strategies that harness the natural capacity of the adaptive immune system to eliminate cancer cells. These strategies include treatment with IL-2 [14], therapeutic monoclonal antibodies [15], inhibitors against CTLA-4 and PD-1 [16,17], oncolytic viruses [18] or the chimeric antigen receptor (CAR) T cell therapy [19]. Certain cancer immunotherapies, including checkpoint inhibitors and adoptive cell transfers, have achieved efficient antitumor responses in patients with a wide range of cancers, including melanoma, renal cell carcinoma, non-small cell lung cancer and Hodgkin lymphoma, transforming their clinical outcomes. Checkpoint inhibitors are therapeutic antibodies that inhibit checkpoint proteins in the cell surface of T cells, such as CTLA-4 and PD-1. Checkpoint proteins limit antitumor immune responses and their inhibition awakens T cells to respond to tumors. Adoptive cell transfer strategies are based on engineering autologous cells from cancer patients and reinfusing them into the same patients where they respond against their tumors. In CAR T cell transfer strategies, T cells are engineered to express a chimeric antigen receptor that recognizes a tumor antigen, enabling the destruction of tumor cells by the reinfused engineered T cells. However, these therapies are effective in only a subset of patients and not in others. Most patients do not benefit from treatment (primary resistance), and some responders relapse after a period of response (acquired resistance) [20]. While CAR T cell treatment to treat pediatric acute lymphoblastic leukemia showed great success, this strategy showed limited efficiency against solid tumors, most likely due to the immunosuppressive tumor microenvironment [21,22]. In addition, some patients that respond to the current therapies develop undesired secondary effects following the treatments, including autoimmune symptoms [23,24]. These facts imply the need to identify additional targets to develop improved cancer immunotherapy strategies for those patients that do not respond or respond poorly to the current treatments, and to reduce secondary effects.

Tumor immunology research has predominantly focused on protein-coding genes, that represent less than 2% of the human genome. During decades, the remaining 98% of the genome that is not translated into proteins was considered “junk” DNA. The recent development of new sequencing methods has revealed that more than 70% of the mammalian genome is transcribed into non-coding RNAs (ncRNAs) [25]. Many ncRNAs have been implicated in cancer development and progression playing both oncogenic or tumor suppressor roles in tumor cells [26,27,28]. Importantly, many ncRNAs regulate the development and function of the immune system [29,30,31,32] suggesting that, in addition to their roles in tumor cells, they might also play central roles in orchestrating antitumor immunity. Here, we review the existing studies that have examined the role of ncRNAs in the specific context of adaptive antitumor responses and discuss their potential future clinical application to advance cancer treatment.

## 2. Non-Coding RNAs as Regulators of Gene Expression

### 2.1. Non-Coding RNAs

NcRNAs are classified based on the length of their nucleotide sequence into small non-coding RNAs (sncRNAs, 18–200 nucleotides) and long non-coding RNAs (lncRNAs, >200 nucleotides) [33]. SncRNAs are further divided into functional and regulatory RNAs. Functional or housekeeping RNAs, including ribosomal RNAs (rRNAs), small nuclear RNAs (snRNAs) and transfer RNAs (tRNAs), participate in ribosome composition and messenger RNA (mRNA) splicing and translation. Functional RNAs are often chemically modified by small nucleolar RNAs (snoRNAs) [34]. Regulatory RNAs regulate gene expression at the epigenetic, transcriptional, and post-transcriptional levels. The most characterized small regulatory RNAs are microRNAs (miRNAs), small interfering RNAs (siRNAs) and PIWI-interacting RNAs (piRNAs) [35]. LncRNAs are emerging as additional regulators of gene expression. Finally, although initially considered as non-functional by-products of the splicing process, circular RNAs (circRNAs) have recently been described as another type of regulatory RNA [36]. So far, only certain types of regulatory ncRNAs, specifically miRNAs and lncRNAs, have begun to be analyzed for their roles in the immune responses to cancer.

### 2.2. MicroRNAs

**Figure 1:**
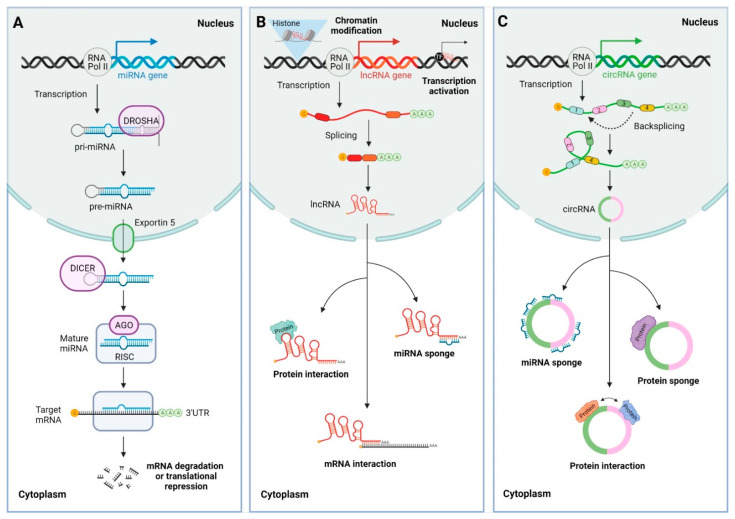
Biogenesis and functions of microRNAs (miRNAs), long non-coding RNAs (lncRNAs) and circular RNAs (circRNAs). (**A**) MiRNAs are transcribed from genomic DNA by RNA polymerase II as primary miRNAs (pri-miRNAs). The pri-miRNAs are processed in the nucleus by Drosha producing precursor miRNAs (pre-miRNAs) which are exported to the cytoplasm by exportin 5. The pre-miRNAs in the cytoplasm are processed by Dicer, which cleaves the hairpin structure and produces a miRNA duplex. The miRNA duplex is loaded to Argonaute (AGO), which binds the guide strand and forms the RNA-induced silencing complex (RISC). The mature miRNAs guide the RISC complex to bind their cognate sites in the 3′ untranslated regions (3′UTR) of the target messenger RNA (mRNA) through imperfect base pair complementarity and induce its translational repression or degradation, thus exerting post-transcriptional gene regulation. (**B**) LncRNAs are transcribed from DNA by RNA polymerase II, similarly to miRNAs. Some lncRNAs undergo splicing and are 5′ capped and 3′ polyadenylated. LncRNAs function in the nucleus inducing chromatin modification and regulating cis and trans gene expression interacting with transcription factors (TFs). In addition, lncRNAs can exit the nucleus and exert different roles in the cytoplasm functioning as miRNA sponges and interacting with proteins and mRNAs to induce or repress their translation. (**C**) CircRNAs are transcribed from DNA by RNA polymerase II and are generated by backsplicing of exon or intronic regions. CircRNAs exit the nucleus and may function as miRNA and protein sponges or as scaffolds for protein interaction.

MiRNAs are small RNAs ∼22 nucleotides in length. They are endogenously encoded and transcribed by RNA polymerase II as primary miRNAs (pri-miRNAs) with hairpin loop structures. Pri-miRNAs are then processed by the RNAse III enzyme Drosha into precursor miRNAs (pre-miRNAs), which are exported by exportin 5 from the nucleus to the cytoplasm and further processed by the RNAse III Dicer to eliminate the hairpin structure, resulting in a miRNA duplex. This miRNA duplex is loaded to an Argonaute (AGO) protein, with only one guide strand remaining associated with AGO and additional proteins that comprise the RNA-induced silencing complex (RISC) (Figure 1A). The mature miRNA in the RISC complex generally pairs through imperfect sequence complementarity with its cognate binding sites in the 3′UTR of target mRNAs, inducing their degradation or translational repression and, thus, resulting in downregulation of gene expression. A major determinant in this recognition process is the exact pairing of the so-called seed region of six to eight nucleotides at the 5′ end of the miRNA [37,38]. Approximately 2500 human miRNAs have been identified and many of them have been linked to the development and/or progression of a large variety of cancer types both as oncomiRs and tumor suppressor miRNAs. In addition, miRNAs perform essential roles in the immune system by regulating the development and function of immune cells [39,40,41]. An estimated 25–40% of miRNA precursors are located in the proximity (<10 kb) of other miRNA precursors, forming miRNA clusters. Most miRNA clusters are transcribed into single polycistronic primary transcripts (pri-miRNAs) and cleaved by Drosha into individual hairpins (pre-miRNAs), which are then processed by Dicer to produce mature miRNAs. An example is the miRNA-17-92 cluster, also known as oncomiR-1, composed of six mature miRNAs belonging to four miRNA subfamilies (miR-17, miR-18, miR-19 and miR-92 subfamilies), with each subfamily defined by a common seed sequence [42]. 

### 2.3. Long Non-Coding RNAs

LncRNAs comprise diverse ncRNAs longer than 200 nucleotides that can be classified according to their location with respect to protein-coding genes and enhancer regulatory elements into long intergenic ncRNAs (lincRNAs), long intronic ncRNAs, antisense RNAs and enhancer RNAs (eRNAs) [36,43]. They are mostly transcribed by RNA polymerase II and undergo splicing, capping of the 5′ ends and polyadenylation of their 3′ ends (Figure 1B). In contrast, eRNAs are generally transcribed bidirectionally from active enhancers and undergo capping, but not splicing or polyadenylation. A recent study estimated the presence of more than 58,000 human lncRNAs [44]. LncRNAs function through modular domains that interact with DNA, RNA or proteins to perform various roles including epigenomic regulation through chromatin modification, and cis and trans regulation of gene expression at transcriptional and post-transcriptional levels. In addition, they indirectly regulate gene expression, acting as miRNA sponges [45,46]. CircRNAs are a special class of covalently closed circular lncRNAs that can range from a hundred to thousands of nucleotides in length. They are generated by backsplicing of precursor RNAs and are not capped or polyadenylated [47] (Figure 1C). CircRNAs function as miRNA and protein sponges, or as regulators of protein function [48]. 

## 3. Regulation of Adaptive Tumor Immunity by Non-Coding RNAs

### 3.1. Immune Responses in Cancer

The first line of defense against pathogens and arising tumors comprises cells of the innate immune system, including neutrophils, macrophages, granulocytes, dendritic cells, mast cells and natural killer cells. Upon tissue homeostasis disruption, tissue-resident macrophages and mast cells locally secrete soluble factors. These include cytokines, chemokines and extracellular matrix remodeling factors that recruit leukocytes from the circulation to the damaged tissue in the process known as inflammation [49,50]. Tumor cells also secrete cytokines and chemokines into the inflammatory microenvironment. At the same time, dendritic cells take up tumor antigens and migrate to lymph nodes, where they present them to cells of the adaptive immune system, composed of T and B lymphocytes. T cells that specifically recognize tumor antigens presented by dendritic cells or other professional antigen presenting cells (APCs) undergo clonal expansion and activation to mount a specific adaptive immune response against the tumor cells [51]. Once the tumor has been eliminated, the inflammatory process ends, and tissue homeostasis is restored. This type of inflammation that limits tumor development is known as acute inflammation. 

Acute inflammatory responses are characterized by the presence of T helper (Th)-1 CD4 T cells, whose differentiation from naïve CD4 T cells is driven by the transcription factor T-bet [52]. These cells provide help to CD8 T cells, exerting a direct effect on the induction of an antitumor cytotoxic response by CD8 cytotoxic T lymphocytes (CTLs). CTLs are critical for immune-mediated tumor cell elimination through their effector molecules granzyme B and perforin. On the other hand, CD4 Th1 cells have an indirect effect on the polarization of tumor-associated macrophages (TAMs) towards a proinflammatory phenotype through the secretion of cytokines such as interferon (IFN)-γ and IL-1 [53,54]. In addition, immunoglobulins (Igs) secreted by B cells can facilitate the recruitment of leukocytes from the innate immune system and the targeted killing of malignant cells [55]. 

However, the inflammatory response necessary to induce antitumor immunity can also promote the growth and spread of neoplastic disease when acute inflammation develops into a chronic state. In 1863, Virchow first postulated that cancer originates from sites of chronic inflammation [56]. Indeed, it is currently well known that up to 20% of cancers are related to chronic infections [57]. When tissues are damaged, compromised cells are killed by inducing cell death pathways. Simultaneously, cell proliferation is promoted to facilitate tissue regeneration and restore tissue homeostasis. If tissue damage persists over time, continuous cycles of cell proliferation and death in microenvironments rich in inflammatory cells that secrete protumoral products can increase neoplastic risk and promote tumor progression [50].

In chronic inflammatory responses, there is a predominance of CD4 Th2 and regulatory T cell (Treg) subsets whose differentiation from naïve CD4 T cells is driven by the transcription factors GATA3 and FoxP3, respectively [52]. These two cell types impair the cytotoxicity of CD8 CTLs. In addition, they release factors into the environment, including IL-4, IL-6, IL-10, IL-13 and TGF-β, which polarize the cells of the innate immune system, including TAMs, towards a protumoral phenotype [54]. Some proinflammatory molecules, such as IFN-γ, tumor necrosis factor (TNF)-α and vascular endothelial growth factor (VEGF), promote the expression of the checkpoint protein programmed death-ligand 1 (PD-L1) that leads to T cell exhaustion [58], which is associated with the impairment of antitumor adaptive immune responses and, thus, favor tumor progression. Checkpoint proteins expressed by T cells include CTLA-4, PD-1, T cell immunoglobulin and mucin domain-containing protein 3 (Tim-3), lymphocyte-activation gene 3 (LAG-3) and T cell immunoreceptor with Ig and ITIM domains (TIGIT), which contribute to T cell exhaustion [59]. The tumor parenchyma is also infiltrated by myeloid-derived suppressor cells that further contribute to the repression of cytotoxic T cell responses [60,61]. Chronically activated B cells can promote the accumulation of innate immune cells in the neoplastic parenchyma through the production of Igs and cytokines. These innate cells secrete growth factors, reactive oxygen and nitrogen species, and angiogenic factors, such as VEGF, into the extracellular environment, favoring tumor progression [55]. 

The important functions of miRNAs during adaptive immune responses have been widely described and reviewed [39]. MiRNAs play key roles in virtually every immune cell process, including development, antigen presentation, cytokine production, innate and adaptive immune cell polarization into different subsets, and T cell activation, effector function and exhaustion, suggesting that they also contribute to the regulation of antitumor immunity. 

### 3.2. Non-Coding RNAs in Immune Responses to Cancer

Dysregulated ncRNA expression has been closely linked with the development or suppression of cancer in humans. However, the role of ncRNAs in tumor progression by their action in the immune system remains understudied. In this section, we compile the existing knowledge on this key component of tumor pathology that, in many cases, determines disease outcome. 

Lung cancer is one of the most frequently diagnosed tumors and the leading cause of cancer-related deaths worldwide [1]. Leidinger et al. performed a miRNome analysis of blood cells of patients with lung cancer and found that miR-21, miR-30b, miR-939, miR-125b, miR-19b, miR-34a, miR-99a, miR-424, miR-31, miR-181a, miR-15b and miR-26a were significantly dysregulated in CD3 T lymphocytes compared to healthy T cells [62], suggesting a potential role for these miRNAs in tumor immunity. A study using the Lewis lung carcinoma (LLC) mouse model, reported that increased expression of miR-15b in CD8 T cells prevents their activation via repressing the production of their effector proteins IL-2 and IFN-γ, as well as their activation-induced cell death (AICD). The authors concluded that the functional inhibition of CD8 T cells by miR-15b impairs antitumor immunity [63]. In addition, Lin et al. analyzed a cohort of advanced lung cancer patients and observed an upregulation of miR-23a in tumor-infiltrating CD8 CTLs that correlated with impaired antitumor potential of such CTLs. They also found that tumor-derived TGF-β directly suppresses CTL immune function by increasing the expression levels of miR-23a. Conversely, functional blocking of miR-23a in human CTLs, using an anti-miR-23a locked nucleic acid (LNA) or retroviral transduction of a miR-23a decoy vector, upregulated granzyme B expression. Treatment of LLC-tumor bearing mice with miR-23a decoy-expressing CTLs impaired tumor progression and significantly reduced tumor burdens. Moreover, tumor pathology examination after 10 days revealed increased expression of the transcription factors T-bet and Eomes and effector molecules IFN-γ and granzyme B in miR-23a-inhibited CTLs, despite CTL persistence within the tumor mass was not affected [64]. This work further supported a role for specific miRNAs in impairing lung cancer antitumor immunity. In addition, previous studies reported that miRNAs could also contribute to protumoral immune responses in the LLC mouse model by functioning as ligands of Toll-like receptors (immune cell receptors that recognize molecular patterns of pathogens) that bind small RNAs, although whether this mechanism directly regulates adaptive immune cells remains to be analyzed [65].

Conversely, both protumoral and antitumoral roles for ncRNAs have been described in the context of colorectal cancer (CRC), the third most deadly cancer globally, whose incidence is steadily rising [1]. A study from Yu et al. reported increased miR-491 expression in splenic CD8 T cells from M-38 colorectal tumor-bearing mice compared to their control counterparts. They also showed that miR-491 overexpression in CD8 and CD4 T cells limits their proliferation, induces apoptosis, and decreases the production of the effector molecules IFN-γ by CD8 T cells in vitro. The authors proposed a model in which tumor-derived TGF-β promotes the upregulation of miR-491 in CD8 T cells which, in turn, decreases the expression of Bcl-xL, TCF-1 and CDK4 and dampens their antitumoral function [66]. On the contrary, a role in tumor suppression has been reported for miR-146a using control or miR-146a knockout murine models of colorectal carcinoma induced in the context of colonic inflammation and spontaneous CRC. This study found that miR-146a-deficient mice were susceptible to both colitis-associated and sporadic CRC. Mechanistic studies partially attributed this effect to a miR-146a-mediated limitation of tumorigenic IL-17 secretion by CD4 T cells. In addition, miR-146a mimics or small molecule inhibition of selected targets ameliorated colonic inflammation and CRC [67]. Regarding lncRNAs, a recent study suggested an antitumor effect for lncRNA GM16343. Specifically, the authors proposed that increased expression of lncRNA GM16343 in CD8 T cells potentiated their capacity to produce IFN-γ and inhibited tumor growth in a xenograft mouse model of colon cancer [68]. 

MiR-21 has been shown to mediate T cell antitumor responses in two distinct mouse models of hepatoma and fibrosarcoma via targeting the Pten (phosphatase and tensin homolog) signaling pathway, further supporting the function of miRNAs in promoting antitumor immunity. Systemic loss of miR-21 favored tumor progression with weakened CD4 and CD8 T cell antitumor responses characterized by impaired T cell proliferation and decreased IFN-γ and IL-2 secretion [69]. Another study that examined the roles of ncRNAs in hepatocellular carcinoma (HCC) suggested a tumor-promoting role for CD8 T cell-expressed lncRNA NEAT1. Specifically, they found that NEAT1 and Tim-3 were upregulated in peripheral blood mononuclear cells (PBMCs) of patients with HCC compared with healthy subjects. Downregulation of NEAT1 in human CD8 T cells impaired their apoptosis and enhanced their cytotoxic activity through a miR-155/Tim-3 pathway. Finally, silencing NEAT1 in murine CD8 T cells impaired tumor growth in a transplantable mouse model of HCC [70]. In addition, lnc-Tim3 was found to be upregulated in tumor-infiltrating CD8 T cells of HCC patients, and negatively correlated with IFN-γ and IL-2 production. The authors attributed a role for lnc-Tim3 in stimulating CD8 T functional exhaustion and survival of these cells by specifically binding to Tim-3 and blocking its interaction with Bat3. This suppressed downstream signaling, led to nuclear localization of Bat3, and enhanced the transcriptional activation of anti-apoptosis genes including MDM2 and Bcl-2 [71]. 

Consistently with other solid tumors, miRNAs are aberrantly expressed in melanoma and exert diverse regulatory roles in tumor immune responses. Expression of miR-23a was shown to inhibit cytotoxic CD8 T cell-mediated antitumor responses in a mouse model of melanoma by directly targeting the transcription factor Blimp-1 and downstream effector molecules granzyme B, T-bet and IFN-γ [64]. On the other hand, multiple studies reported that the levels of CD8 T cell-expressed miR-155 associate with optimal T cell receptor (TCR)-induced activity of tumor-infiltrating CD8 T cells (TILs), increased IFN-γ responses in both CD4 and CD8 T cells through a mechanism involving Ship1 repression, and to a better control of tumor growth [72,73,74,75]. One of these reports also established an opposing protumoral role for miR-146a, as evidenced by reduced tumor growth and enhanced IFN-γ production by both CD4 and CD8 T cells from miR-146a knockout tumor-bearing mice [74]. In another study, Jiang et al. showed that loss of the miR-17-92 cluster in CD4 T cells impaired their Th1 functional capacity and reduced their ability to provide help to CD8 T cells in murine melanomas. They functionally dissected the cluster and identified miR-17 and miR-19b as the key regulators of Th1 responses. These miRNAs promoted the proliferation of Th1 cells, protected them from AICD, supported IFN-γ production, and suppressed inducible Treg (iTreg) differentiation. Mechanistically, the authors identified Pten as the functional target of miR-19b, and TGFRII and CREB1 as the targets of miR-17 [76]. Additionally, increased expression of miR-28 on murine T cells decreased their exhaustion state in B16-F10 tumor-bearing mice, as shown by the recovery of their capacity to secrete IL-2 and TNF-α [77]. 

A role for miR-149-3p in T cell exhaustion has also been reported in the context of breast cancer. Zhang et al. recently showed that treatment of CD8 T cells with a miR-149-3p mimic reverses their exhausted phenotype and increases their activation as indicated by their IL-2, IFN-γ and TNF-α production and increased cytotoxic capacity in a murine breast cancer model [78]. In addition, miR-155 has also been shown to enhance T cell-mediated antitumor responses in the EL4 mouse model of thymic lymphoma by increasing their IFN-γ production [74].

Given that miR-124 is absent in all grades and pathologic types of gliomas, Wei et al. examined the effect of increasing the levels of this specific miRNA in glioma cancer stem cells (gCSC). They found that miR-124a inhibited the STAT3 pathway and reversed the gCSC-mediated immunosuppression of T cell proliferation and induction of Tregs. Furthermore, T cells from immunosuppressed glioblastoma patients treated with miR-124 upregulated IL-2, IFN-γ and TNF-α, which enhanced their effector response. Finally, increased miR-124 levels potentiated T cell-mediated responses in the tumor microenvironment and drastically reduced tumor burden in glioma mouse models [79]. Another study from the same group focused on the role of miR-138, a miRNA with predicted binding sites on both CTLA-4 and PD-1, in antitumor immunity. The authors reported that miR-138 treatment of GL261 gliomas in immune-competent, but not in immune-deficient (nude), mice induced a significant tumor regression, increased mice survival and decreased the number of intratumoral Tregs as well as CTLA-4 and PD-1 expression. The effect of this treatment was also lost in CD4 or CD8 T cell-depleted mice, and a potential direct suppressive effect of miR-138 treatment on glioma cells was excluded [80].

**Figure 2:**
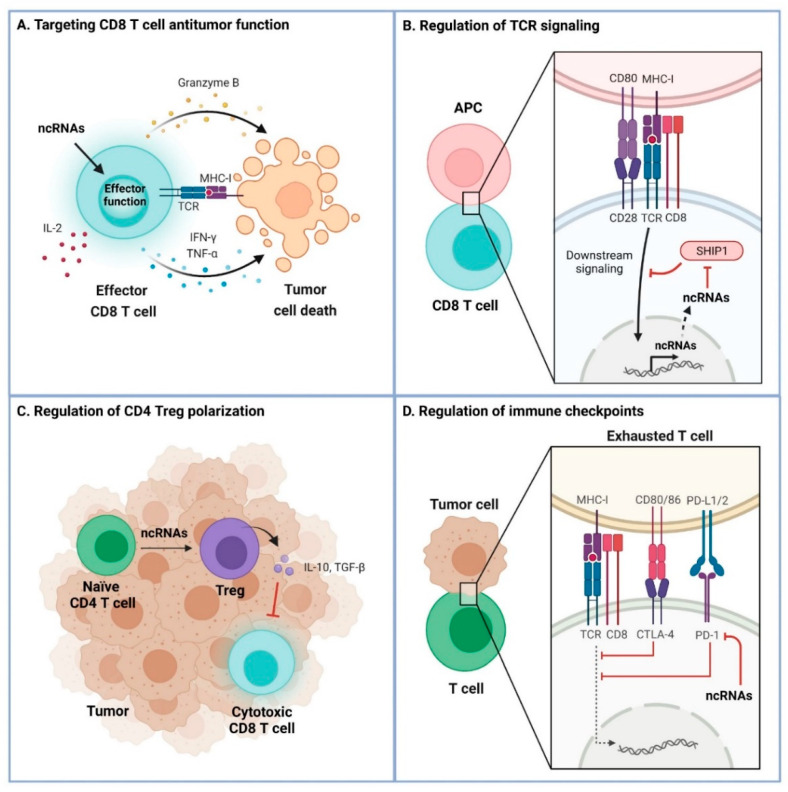
Molecular regulatory mechanisms of ncRNA in tumor immunology. (**A**) Targeting CD8 T cell antitumor function. CD8 T cells recognize tumor cells and exert cytotoxic functions upon engagement of the T cell receptor (TCR) and the antigen-derived peptide loaded on major histocompatibility type I molecules (MHC-I) on tumor cells. NcRNAs regulate the antitumor function of CD8 T cells, characterized by secretion of IL-2, IFN-γ, TFN-α and granzyme B. (**B**) Regulation of TCR signaling. Downstream signaling induced upon TCR engagement promotes the expression of ncRNAs. NcRNAs can control TCR signaling by targeting phosphatase SHIP1, a negative regulator of TCR signaling, thereby increasing TCR signaling. (**C**) Regulation of CD4 Treg polarization. NcRNAs are involved in the differentiation of naïve CD4 T cells into regulatory T cells (Tregs) which secrete TGF-β and IL-10 that inhibit cytotoxic CD8 T cells in the tumor microenvironment. (**D**) Regulation of immune checkpoints. NcRNAs also control the T cell exhaustion state by directly targeting checkpoint proteins.

Overall, ncRNAs have been shown to play both antitumoral and protumoral roles by their action in the immune system in a tumor setting (Table 1). The main molecular mechanisms that the ncRNAs use to regulate tumor immunology are summarized in Figure 2. These mechanisms include targeting the CD8 T cell antitumor function by modulating the expression of IL-2, IFN-γ and TNF-α, altering TCR signaling, controlling the induction of Treg populations, and regulating immune checkpoint proteins including PD-1, CTLA-4 and Tim-3. 

## 4. Conclusions and Future Prospects

The clinical potential of the immune system to eliminate tumors has been demonstrated by the success of certain cancer immunotherapies in some patients. A major advantage of cancer immunotherapy is the durability of specific antitumor responses that result in long-term survival for a subset of patients. This is most likely due to the immunological memory of adaptive immunity. Furthermore, the immune system can recognize and clear distant metastasis in addition to the primary tumor. However, many patients still do not respond to the treatments or develop resistance. Expanding clinical benefit to most patients requires a better understanding of the mechanisms that lead to effective antitumor responses. 

The recent advances in our understanding of the role of ncRNAs in the context of tumor immunology pave the way to future research in this field, providing the opportunity to expand our basic knowledge and potentially identify new targets for cancer immunotherapy. So far, the role of certain selected individual ncRNAs has been analyzed through genetic approaches as described above. Future studies based on unbiased in vivo experimental approaches that examine the role of large ncRNA libraries in specific cancer contexts should shed new light into the immunobiology of cancer. These systematic approaches enable the identification of target candidates that would be missed through traditional studies, including genes with previously unknown functions. Among the different ncRNAs that have been annotated, miRNAs are attractive targets to enhance antitumor immunity due to their intrinsic capacity to control cell functions quickly and efficiently through the simultaneous downregulation of a network of cell signaling pathways that control cell proliferation, apoptosis and effector functions, among other processes [37,41]. In addition, their small size allows them to inhibit mRNA targets inside tissues without the need for complex delivery vehicles. MiRNA therapeutics could potentially be used in combination with CAR T cell strategies to improve their function in the context of solid tumors, or to boost endogenous T cell responses. Another advantage of miRNAs from a therapeutic perspective is that some of their protein-coding target genes might have ideal properties for therapeutic intervention. Thus, establishing the targetomes of miRNAs that control antitumor responses in specific immune cell subsets might lead to the identification of new biomarkers and/or relevant druggable targets, including immune checkpoint proteins, to improve the efficiency of current cancer immunotherapies so that they may benefit a greater number of patients.

Studies using miRNA mimics and inhibitors in mice have shown therapeutic effects in different diseases [81,82,83,84,85,86]. In addition, multiple miRNA-based compounds are currently undergoing phase 2 clinical trials, including Miravirsen (Santaris Pharma), a treatment for hepatitis C virus infection. A completed phase 2 study for Miravirsen supports the feasibility of miRNA-based therapeutic approaches in humans. In that study, the compound showed high barrier to viral resistance, favorable tolerability and low propensity for drug interactions [87]. Many cancer clinical trials based on miRNAs are ongoing [88], but none of them has focused on the modulation of patients’ adaptive immune system so far. As our knowledge regarding the relationship between ncRNAs and tumor immunology evolves, it should be interesting to test the possibility of using ncRNA to boost antitumor immunity in future clinical trials. Importantly, small chemical inhibitors for some of the identified protein-coding target genes might already be available. In addition, therapeutic antibodies targeting relevant cell surface proteins identified through these studies could be developed.

In summary, some studies showing roles for certain ncRNAs in promoting or restricting antitumor immunity have been developed during the past few years, still covering a very minor fraction of our non-coding genome. Systematic unbiased studies in different cancer types are required to expand our integrated knowledge of how regulatory ncRNAs impact antitumor immunity. A deeper understanding of the functional mechanisms of all ncRNAs on adaptive immune cells could broaden the clinical applicability of ncRNAs in tumor immunotherapy.

## Figures and Tables

**Table 1:** Overview of ncRNA functions in the context of tumor immunology.

ncRNA	Cell Subset	Function	Cancer	References
miR-15b	CD8 T cells	↓ AICD ↓ IFN-γ/IL-2 production	Lung	[63]
miR-23a	CD8 T cells	↓ Cytotoxicity ↓ IFN-γ production ↓ Blimp-1, Eomes, T-bet, Granzyme B	Lung Melanoma	[64]
miR-146a	T cells	↓ Antitumor responses ↓ IFN-γ production	Melanoma	[74]
miR-491	T cells	↓ Proliferation ↓ IFN-γ production ↑ Apoptosis ↓ Bcl-xL, TCF-1, CDK4	Colorectal	[66]
Lnc NEAT1	CD8 T cells	↓ Cytotoxicity ↑ Tim3	Hepatocarcinoma	[70]
Lnc-Tim3	CD8 T cells	↑ Exhaustion ↓ IL-2/IFN-γ production Binds Tim-3 and blocks interaction with Bat3	Hepatocarcinoma	[71]
miR-17-92	T cells	↑ Th1 function ↑ CD4 T cell help to CD8 T cells ↓ iTreg induction ↓ Pten, TGFRII, CREB1	Melanoma	[76]
miR-21	T cells	↑ Antitumor responses ↑ Proliferation ↑ IFN-γ/IL-2 production ↓ Pten signaling	Hepatoma Fibrosarcoma	[69]
miR-28	T cells	↓ Exhaustion ↑ IL-2/TNF-α production	Melanoma	[77]
miR-124	T cells	↑ Proliferation ↓ Treg induction ↑ IFN-γ/IL-2/TNF-α production ↓ STAT3	Glioma	[79]
miR-138	T cells	↓ Treg induction ↓ CTLA-4, PD-1	Glioma	[80]
miR-146a	CD4 T cells	↓ IL-17 production	Colorectal	[67]
miR-149-3p	CD8 T cells	↓ Exhaustion ↑ Activation ↑ Cytotoxicity ↑ IFN-γ/IL-2/TNF-α production	Breast	[78]
miR-155	T cells	↑ Antitumor responses ↑ IFN-γ production ↓ Ship1	Melanoma Thymic lymphoma	[72,73,74,75]
Lnc GM16343	CD8 T cells	↑ IFN-γ production	Colon	[68]

↑: increased, ↓: reduced.
